# Octane-Assisted Reverse Micellar Dyeing of Cotton with Reactive Dyes

**DOI:** 10.3390/polym9120678

**Published:** 2017-12-06

**Authors:** Alan Yiu-lun Tang, Cheng-hao Lee, Yanming Wang, Chi-wai Kan

**Affiliations:** 1 Institute of Textiles and Clothing, The Hong Kong Polytechnic University, Hung Hom, Kowloon, Hong Kong, China; alan.yl.tang@connect.polyu.hk (A.Y.-l.T.); amyymwang@gmail.com (Y.W.); 2 Department of Applied Biology and Chemical Technology, The Hong Kong Polytechnic University, Hung Hom, Kowloon, Hong Kong, China; chenghao.lee@polyu.edu.hk

**Keywords:** cotton fibre, non-ionic surfactant, octane, reverse micelle, reactive dye, colour matching

## Abstract

In this study, we investigated the computer colour matching (CCM) of cotton fabrics dyed with reactive dye using the octane-assisted reverse micellar approach. The aim of this study is to evaluate the colour quality and compare the accuracy between CCM forecasting and simulated dyeing produced by conventional water-based dyeing and octane-assisted reverse micellar dyeing. First, the calibration of dyeing databases for both dyeing methods was established. Standard samples were dyed with known dye concentrations. Computer colour matching was conducted by using the colour difference formula of International Commission on Illumination (CIE) L*a*b*. Experimental results revealed that the predicted concentrations were nearly the same as the expected known concentrations for both dyeing methods. This indicates that octane-assisted reverse micellar dyeing system can achieve colour matching as good as the conventional water-based dyeing system. In addition, when comparing the colour produced by the conventional water-based dyeing system and the octane-assisted reverse micellar dyeing system, the colour difference (Δ*E*) is ≤1, which indicates that the reverse micellar dyeing system could be applied for industrial dyeing with CCM.

## 1. Introduction

Dyeing textile products has been regarded as a significant consumer of water and producer of effluents [1,2] and the addition of large amounts of salts is required in water-based reactive dye dyeing processes [3]. In the past, several attempts have been made to reduce effluent discharge such as (i) low-salt or salt-free dyeing [4,5,6]; (ii) dyeing with natural mordants [7,8,9]; (iii) reusing wastewater as an alternative water source for dyeing [10]; (iv) supercritical carbon dioxide dyeing [11,12,13,14,15]; and (vi) clay nano-adsorbent treatment [16]. Among different methods, use of reverse micelles has been considered for energy saving, ecological friendliness and safety in the working environment [17,18,19,20]. The reverse micelles, self-assembled by a certain surfactant in a non-polar medium, are nanoscale spherical aggregates [19,20]. They have the ability to solubilize small amounts of water in their interior regions, providing a stable aqueous micro-environment (water-pool) in a non-aqueous medium such as organic solvent. By using the reverse micelle approach in textile dyeing, a salt-free dyeing process can be achieved so that the salt concentration in the effluent discharge can be substantially reduced. Replacing water with the use of solvent as the dyeing medium can also greatly reduce water consumption, which can alleviate the problem of shortages in water supply, reduce the need for water purification and minimize the cost of effluent treatment since the solvent and other auxiliaries can be recycled by a simple method [18]. Further comparison between water-based conventional dyeing and reverse micelle dyeing is listed in Table 1.

Attempts have been made to dye cotton with reverse micelles in a non-aqueous medium [21,22,23]. Experiments have also been conducted for dyeing cotton in heptane and octane solvent using polyethylene glycol (PEG) based nonionic reverse micelles [18]. Although many of the studies have been done to develop non-aqueous dyeing systems for cotton fabrics, they have focused solely on dyeing with a single colour or primary colours. In everyday dyeing production, different colours are produced with different combinations of primary colours (i.e., red, yellow and blue) and the appearance of the textile products is mostly a mixture of colours. Colour matching is thus of great importance in industrial applications. Computer colour matching (CCM) is used in the dyeing industry which combines the use of computer and colorimetry knowledge for the realization of colour measures and recipe prediction [24]. CCM involves the application of the Kubelka–Munk Theory and colour difference formulae, for recipe formulation. In recent years, CCM has been used comprehensively in textile dyeing and printing [25,26,27]. However, the feasibility of applying CCM on reverse micellar dyeing of cotton has not yet been studied intensively [28].

In our previous study, we used decamethylcyclopentasiloxane (D5) for reverse micellar dyeing of cotton with reactive dyes and CCM in this D5 reverse micellar dyeing is proved to be feasible [28]. However, the cost of D5 is relatively high and thus, in this study, octane (C_8_H_18_), which is commercially available and gives a higher colour yield when applied in reverse micelle cotton reactive dyeing, [18] was used as the non-aqueous medium for the reverse micellar dyeing of cotton fabrics. There are several tasks in this study: (a) establishing a dyeing database for both conventional water-based dyeing and octane-assisted reverse micellar dyeing for calibration purposes; (b) dyeing standard samples with known concentrations of reactive dyes using conventional water-based dyeing and octane-assisted reverse micellar dyeing methods; (c) conducting computer colour matching to generate dyeing recipes with the use of different colour formulae; and (d) comparing the differences between conventional water-based dyeing and octane-assisted reverse micellar dyeing.

## 2. Experimental Details

### 2.1. Materials and Reagents

Commercial, ready-for-dyeing, 100% cotton, interlock-knitted fabric (40 wales per inch × 40 courses per inch) was used. Poly(ethylene glycol) (12) tridecylether (C_13_H_27_(OCH_2_CH_2_)_12_OH) (PEG) was used as the nonionic surfactant. Octane was used as organic solvent and *n*-octanol was used as co-surfactant in the dyeing process. Sodium chloride (NaCl) was used in conventional water dyeing. Soda ash (Na_2_CO_3_) was used as the colour fixation agent. Surfactant, solvent, co-surfactant, sodium chloride and soda ash were of reagent grade. Three reactive dyes, Levafix Yellow CA, Levafix Red CA and Levafix Blue CA (Dystar, Shanghai, China) were directly used without further purification.

### 2.2. Pre-Cleaning of Cotton Fabric

The cotton fabric was first cleaned with a solution containing 2 g/L soda ash and 2 g/L soap with a thermal-control shaker (Jeio Tech, Seoul, Korea) at 90 °C for 30 min. Then, the cleaned fabric was thoroughly rinsed with cold water and dried at room temperature. The cleaned fabric was then conditioned at a relative humidity of 65 ± 2% and 20 ± 2 °C for at least 24 h before further experiments.

### 2.3. Dyeing Cotton in Water

Dyeing was conducted at a liquor-to-goods ratio of 50:1. Three reactive dyes, Levafix Yellow CA, Levafix Red CA and Levafix Blue CA, were used with five concentrations (0.1, 0.5, 1.5, 2.5 and 3.5% owf). The dyeing recipe for different dye concentrations was as shown in Table 2. The amount of NaCl added was based on the concentration of reactive dye used (% owf), as listed in Table 2.

**Figure 1:**
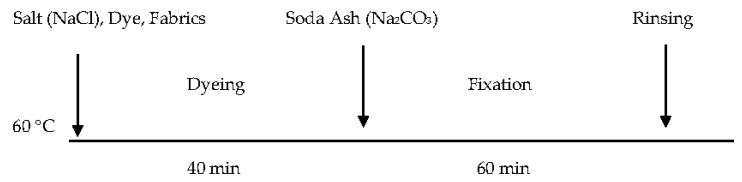
Dyeing profile of cotton in water.

Figure 1 shows the profile for dyeing cotton fabric in water [28]. The cotton fabric was first immersed in the dye liquor and the dyeing process was carried out in a shaking water bath. The dyeing process was conducted at 60 °C for 40 min. After that, the corresponding amount of Na_2_CO_3_ (according to Table 2) was added into the dye liquor for dye fixation. The fixation process was conducted at 60 °C for 60 min. After dyeing, the dyed fabric was washed in a soap solution (2 g/L) and rinsed for 15 min at 60 °C. In order to remove the unfixed dyes, the cleaned cotton fabric was then rinsed with water, air-dried and conditioned at a relative humidity of 65 ± 2% and 20 ± 2 °C for at least 24 h prior to further experiments.

### 2.4. Dyeing Cotton with the Reverse Micellar System

A series of reverse micelle were prepared by a simple injection method at room temperature according to [18,28]. The non-ionic surfactant (PEG) and co-surfactant (1-octanol) were first premixed (with agitation). The surfactant to co-surfactant ratio was 1:8 in mole ratio. The surfactant/co-surfactant mixture was then dissolved in an octane solvent to obtain an organic surfactant solution for facilitating the self-assembly of reverse micelle. The solvent volume to cotton weight ratio was 8:1. Three reactive dyes, Levafix Yellow CA, Levafix Red CA and Levafix Blue CA, were used as received, with further purification for five concentrations separately [18,28]. A controlled amount of reactive dye aqueous solution (0.5 mL) was then applied into the reverse micellar system dropwise. After injection of the dye solution, the mixtures were vigorously stirred for 2 min until a well-dispersed solution with reverse micelle encapsulated reactive dye was obtained.

**Figure 2:**
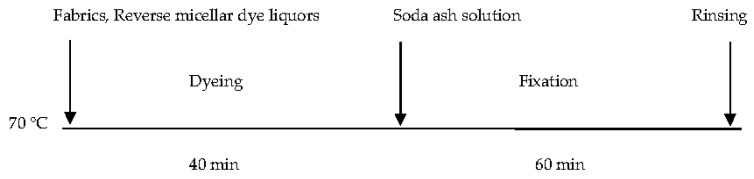
Dyeing profile of cotton in the reverse micellar system using octane.

The dyeing of cotton with the reverse micellar system was conducted at a surfactant to water ratio of 1:20 in mole ratio. The reverse micellar dye liquors were prepared without the addition of electrolytes (NaCl), by adding the corresponding amount of surfactant, co-surfactant, dye solutions, octane and deionized water. The dyeing profile of reverse micellar dyeing in octane was as shown in Figure 2. The cotton fabric was first immersed in the reverse micellar dye liquor and then put in a shaking water bath. The dyeing process was conducted at 70 °C for 40 min. Then, the corresponding amount of soda ash (according to Table 3) was added into the dye liquor for dye fixation. The fixation process was conducted at 70 °C for 60 min. Soaping was conducted after the fixation process by immersing the dyed cotton fabric in soap solution (2 g/L) and rinsing lasted for 15 min at 60 °C. The rinsed cotton fabric was then rinsed with water, air-dried and conditioned at relative humidity of 65 ± 2% and 20 ± 2 °C for at least 24 h prior to further experiments.

### 2.5. Establishment of Calibration Curves

Colour yields of dyed fabrics (both water and reverse micellar dyed) were measured by Color Eye 7000A Spectrophotometer (X-Rite, Singapore). The reflectance and *K*/*S* values were measured and the values could be stored in the computer of the spectrophotometer for further algorithmic processing. The *K*/*S*_sum_ value was obtained by the summation of the *K*/*S* values measured at wavelengths between 400 and 700 nm. The face of the dyed fabrics was measured under specular, large aperture (30 mm), illuminant D_65_ and standard observer of 10°. The opacity of the fabric was assured by folding the fabric two times. The colour yield, expressed as *K*/*S*_sum_ value, was calculated by Equation (1) from the individual *K*/*S* values from wavelength 400 to 700 nm with 10 nm intervals within the visible spectrum. The higher the *K*/*S*_sum_ value, the greater the dye uptake and the better the colour yield is [18,28].

*K*/*S* = (1 − *R*)^2^/2*R* (1)

where *K* is the absorption coefficient, depending on the concentration of colourant, *S* is the scattering coefficient, caused by the dyed substrate and *R* is the reflectance of the coloured sample.

After obtaining *K*/*S*_sum_ values of different dye concentrations, i.e., 0.1, 0.5, 1.5, 2.5 and 3.5%, of the dyed fabrics (termed as batch sample), calibration curves constructed with a plot of *K*/*S*_sum_ value versus concentration could be obtained and stored in the computer of the spectrophotometer for further CCM usage.

### 2.6. Simulated Dyeing for Computer Colour Matching

Simulated dyeing with known concentrations of dye was conducted in order to predict the dye concentration based on the calibration curves of both the water-based and reverse micellar dyeing methods. The simulated dyed fabrics were regarded as the standard samples used for computer colour matching. The concentration of dye in the recipe for preparing a standard sample with a mixture of colours was as shown in Table 4. The dyeing was carried out according to the methods mentioned for water-based and reverse micellar dyeing.

### 2.7. Predicting Dye Recipe

After dyeing, *K*/*S*_sum_ values of the standard samples were measured with a Color Eye 7000A Spectrophotometer (X-Rite, Grand Rapids, MI, USA). Based on the calibration curves established in Section 2.5, concentrations of dyes in the standard samples could be predicted through algorithmic calculations using the established calibration curves (obtained in Section 2.5). Then, the known dye concentration and predicted dye concentration in the dye recipe could be compared to find out the accuracy of computer colour matching between the two dyeing methods. If the result of the known dye concentration and predicted dye concentration in the dye recipe is similar, it means the colour of samples dyed by the reverse micellar dyeing method could be predicted by computer colour matching.

### 2.8. Colour Difference

The water-based dyed fabrics (obtained in Section 2.6) were used as the standard fabric and CCM was used for predicting dyeing recipes to reproduce the colour by using octane-assisted reverse micellar dyeing. The CIE L*a*b* values could be obtained by Color Eye 7000A Spectrophotometer (X-Rite, Grand Rapids, MI, USA) and the colour difference (Δ*E*) could be calculated by Equation (2):

(2) $$\Delta E=\sqrt{{\left(\Delta L\right)}^{2}+{\left(\Delta a\right)}^{2}+{\left(\Delta b\right)}^{2}}$$

where *L* is lightness; *a* refers to the colour component of red and green; and *b* refers to colour component of yellow and blue

Δ*L* = *L*_water_ − *L*_octane_; Δ*a* = *a*_water_ − *a*_octane_; and Δ*b* = *b*_water_ − *b*_octane_

## 3. Results and Discussion

### 3.1. Reflectance Values of the Dyed Samples

**Figure 3:**
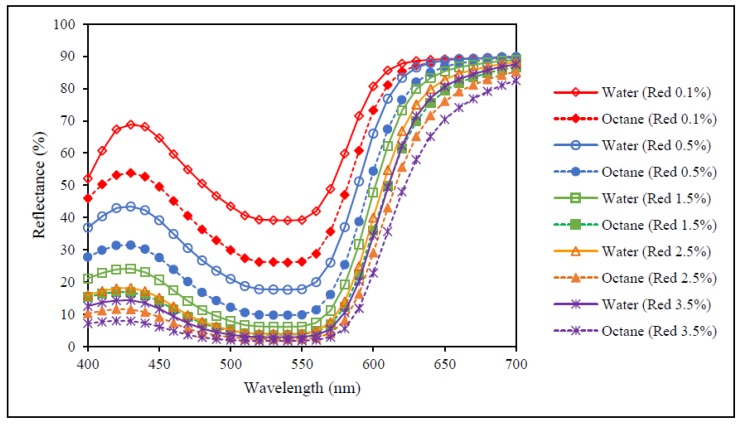
Reflectance curves of red colour.

**Figure 4:**
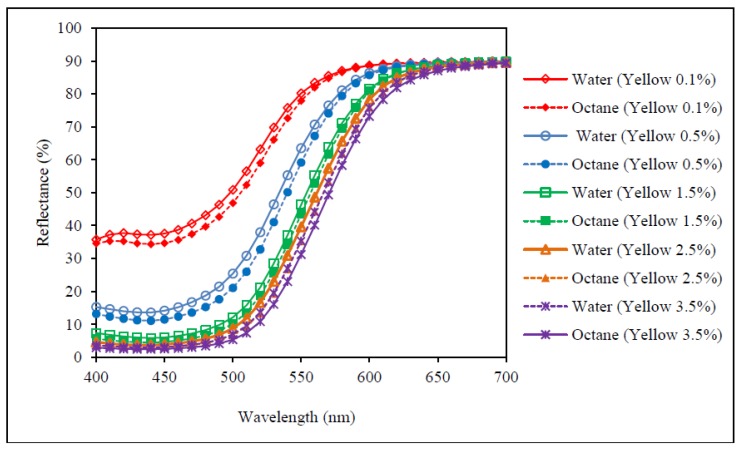
Reflectance curves of yellow colour.

**Figure 5:**
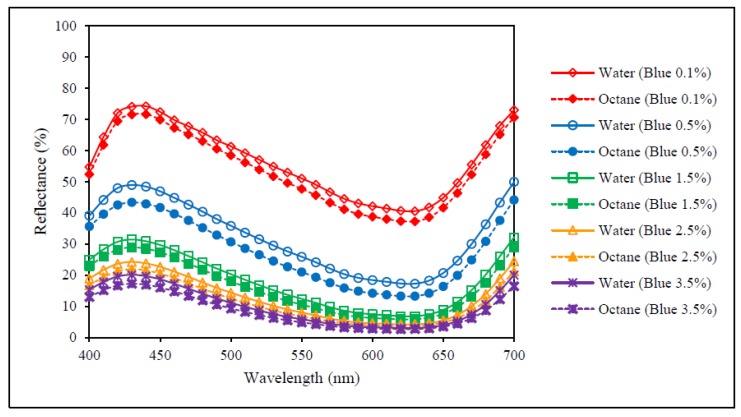
Reflectance curves of blue colour.

**Figure 6:**
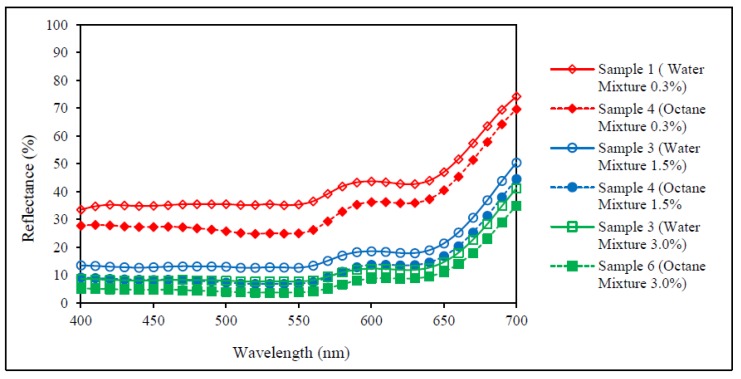
Reflectance curves of colour mixture.

Figure 3, Figure 4 and Figure 5 show the reflectance curves of red, yellow and blue dyed samples (obtained from Section 2.3 and Section 2.4) respectively, while Figure 6 presents reflectance curves of standard samples (obtained from Section 2.6). With reference to Figure 3, Figure 4 and Figure 5, the results show that reflectance values of samples dyed in water are higher than the samples dyed by octane-assisted reverse micellar dyeing at each dye concentration (0.1%, 0.5%, 1.5%, 2.5% and 3.5%). This reveals that the dye absorption in the fibre and the colour yield of the sample dyed in water are lower and the shade appears to be lighter than that of the samples dyed in the octane medium. Among the three primary colours, the difference of reflectance values of the colour red at each dye concentration is most significant, followed by the colour blue and the colour yellow. This indicates that by using the same dye concentration, octane-assisted reverse micellar dyeing could greatly enhance the colour yield of the fabrics [18].

Concerning Figure 6, the results show obviously that reflectance values of the standard samples dyed in water at each dye concentration (i.e., 0.3%, 1.5% and 3.0%) are higher than the standard samples dyed in the octane medium and no overlapping of the reflectance curves is observed in the visible spectrum. This indicates that the dye absorption of the standard samples dyed in octane medium at each dye concentration is higher, and thus achieves a better colour yield in which the shade appears to be darker when compared with the standard samples dyed in a water medium.

**Figure 7:**
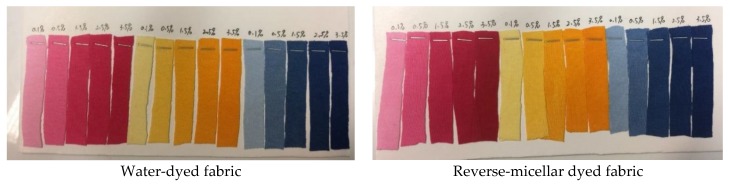
Dyeing results.

Although the reflectance values, as shown in Figure 3, Figure 4, Figure 5 and Figure 6, of the dyed samples are not similar for each dye concentration, the reflectance curves of the samples dyed by conventional water-based and octane-assisted reverse micellar dyeing methods are similar to each other in shape, and no shifting of the peak of the curves is noted. This indicates that the use of octane-assisted reverse micellar dyeing could be an alternative method to achieve electrolyte-free (salt-free) and water-saving dyeing of cotton fibre, as well as allowing computer colour matching (CCM) to be carried out without a chromatic shift. Figure 7 shows visually that octane-assisted reverse micellar dyeing can achieve better dyeability than water-based dyeing with good levelness and there is no obvious shade change in the final colour, which is in good agreement with the reflectance curves.

### 3.2. Linearity of the Calibration Curves

**Figure 8:**
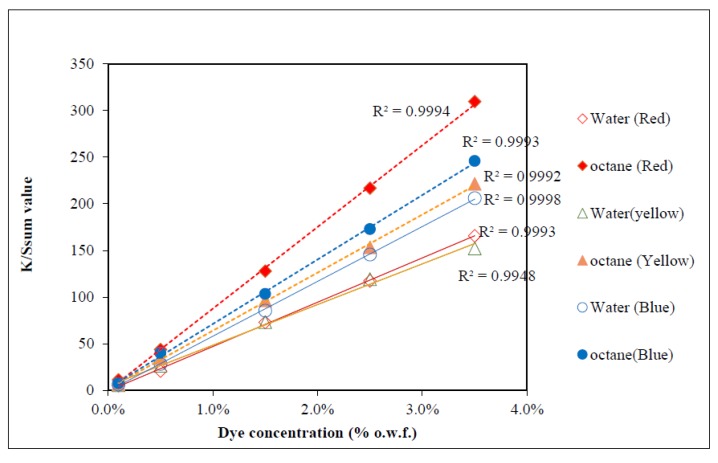
Calibration curves for computer colour matching.

The linearity of the calibration curves is calculated in terms of R-Square (R^2^). The R-Square is a method widely applied for measuring the goodness-of-fit of a regression [29]. The value of R-Square ranges from 0 to 1, where 0 means non-linear and 1 means linear in structure. As revealed from Figure 8, the value of R-Square obtained by conventional water-based dyeing ranges from 0.9948 (yellow) to 0.9998 (blue) while the value of R-Square obtained in the case of octane-assisted reverse micellar dyeing ranges from 0.9992 (yellow) to 0.9994 (red). This revealed that the calibration curves of samples dyed using the conventional water and octane-assisted reverse micellar systems are linear and suitable for subsequent CCM. The linearity of the calibration curves of octane-assisted reverse micellar dyeing is generally better than water-based dyeing.

Figure 8 shows calibration curves of the three primary colours (red, yellow and blue) of the samples dyed in water and an octane-assisted reverse-micellar medium. The calibration curve is a plot of *K*/*S*_sum_ value versus dye concentrations. As presented in Figure 8, the results show that the *K*/*S*_sum_ values of red, yellow and blue colours of the samples dyed using the octane-assisted reverse micellar system are higher than samples dyed in water. It means that the use of the octane-assisted reverse micellar dyeing system could achieve a better colour yield than the conventional water dyeing system. One of the possible reasons is that the use of the reverse micellar system could reduce the ionization effect between reactive dye molecules and the cotton fibre, which improves the swelling of cotton fibres and means that they can accept more dye molecules [17,20]. Another possible reason could be that the octane-assisted reverse micellar dyeing system could minimize the effect of dye hydrolysis because less water is used when compared with conventional water-based dyeing [18], so that more reactive dye molecules could react with the cotton fibre.

### 3.3. Computer Colour Matching (CCM)

Table 5 shows the predicted computer colour matching recipes for standard samples dyed by the conventional water-based method and the octane-assisted reverse micellar dyeing system based on the established calibration curves (obtained in Section 2.5). The results revealed that the reverse micellar dyeing system in octane could have a better recipe prediction than the conventional water-based method. It is observed that the CCM predicted concentrations generated by using the conventional water-based method are lower than known concentrations (i.e., the concentrations used for simulated dyeing in Section 2.6). Whereas the CCM predicted concentrations generated by using the octane-assisted reverse micellar dyeing method are generally higher than the known concentrations. For the predicted values lower than the known concentrations, it can be explained that the dye molecules are insufficient for even distribution within the fabrics, while for samples with higher predicted values than the known concentration, the possible reason is the formation of a dye aggregate which may influence the absorption and scattering of light [28]. However, the difference between known concentration and CCM predicted concentration can also be explained by the linearity of the calibration curves [28]. The higher the linearity of the calibration curves, the higher would be the accuracy of the result and the smaller would be the difference between known and CCM predicted concentration. It is observed that at a higher concentration (3%), the difference between the known and the CCM predicted concentration is greater than that at a lower concentration for both methods, and this is explained by the degree of dye aggregation. At high concentration, the possibility of forming dye aggregates and having insufficient dye molecules distributed within the fabric would be generally higher than at low concentration.

### 3.4. Colour Difference

Colour difference formulae are widely used for quality control, computer colour matching, shade sorting and evaluation of colorfastness. There are two main usages of color difference formulae in computer colour matching: (a) to determine the colorimetric closeness of the computed formula to the target; and (b) to calculate the metameric indices. The International Commission on Illumination (CIE) recommended the use of two colour difference formulae in 1976: CIELAB and CIELUV. Although more advanced formulae such as CIEDE2000, have been developed in recent years, several colour difference formulae are still widely used in industrial applications, such as CIELAB, Hunter Lab, FMC-2, CMC and CIELUV [28].

Within the CIE L*a*b* system, L*a*b* represents lightness, red-green axis and yellow-blue axis respectively while Δ*E* represents, in a quantitative manner, the colour difference between the two specimens (in this case, conventional water-based dyed samples and octane-assisted reverse micellar dyed samples). Under controlled conditions with two objects placed next to each other, the colour difference with a Δ*E* value of 1 or higher could be perceived by the human eye.

Table 6 shows the Δ*E* results of different water-based dyed samples and reverse micellar dyed samples and the results show that the octane-assisted reverse micellar dyed samples could achieve Δ*E* ≤ 1 when compared with conventional water-based dyed samples. These results indicate that the colour difference between samples dyed by the two methods cannot be visually perceived by the human eye. Thus, the results attest the colour quality of the samples produced by the octane-assisted reverse micelle dyeing method. In addition, it indicates that the octane-assisted reverse micellar dyeing process can be applied in industrial dyeing processes using normal colour matching practice with CCM to reproduce colours produced by conventional water-based dyeing.

## 4. Conclusions

CCM, which is a reliable mathematical forecasting model and an objective measurement for the high-accuracy evaluation of colour quality, was used to investigate the shade of the colour mixture produced by water-based and octane-assisted reverse micellar dyeing systems. The experimental results showed that CCM was not only applicable in the conventional water-based dyeing system, but also in the non-aqueous octane-assisted reverse micellar dyeing system. Reflectance values of the dyed samples were measured, and the results showed no chromatic or colour change when octane-assisted reverse micellar dyeing was used. The calibration dyeing databases for both conventional water-based dyeing and octane-assisted reverse micellar dyeing were established. The calibration curve was almost linear with R-Square ranged from 0.9948 to 0.9998, which is suitable for subsequent CCM formulation. The *K*/*S*_sum_ values of octane-assisted reverse micellar dyed samples were higher than that of water-based dyed samples at each dye concentration, indicating that the use of octane-assisted reverse micellar dyeing can achieve better colour yields than that of the conventional water-based method. CCM was conducted by using colour difference formulae and the results revealed that the concentrations predicted by CCM were nearly the same as the known concentrations for both methods. The colour difference between samples dyed by two different methods is presented according to the CIE L*a*b* system and the value of Δ*E* is less than 1. This indicates that octane-assisted reverse micellar dyeing can achieve good colour matching, comparable to conventional water-based dyeing system, in industrial applications.

## Figures and Tables

**Table 1:** Comparison between conventional and reverse micelle dyeing.

Characteristics	Water-Based Conventional Dyeing	Reverse Micellar Dyeing
Color yield	Lower	Higher
Electrolyte addition	Yes	No
Salt content in effluent	Yes	No
Water consumption	Higher	Lower
Effluent production	Higher	Lower
Effluent treatment cost	Higher	Lower
Environmental friendliness	Lower	Higher
Recyclability	Lower	Higher
Effluent treatment techniques	Complex	Simple

**Table 2:** Dye recipe for calibration dyeing.

Dye Concentration (% owf *)	Additives	
	Salt (NaCl, g/L)	Soda Ash (Na_2_CO_3_, g/L)
0.1	10	5
0.5	20	5
1.5	42.5	5
2.5	55	5
3.5	65	5

* % owf = percentage on weight of cotton fabric (g).

**Table 3:** Concentration of soda ash used for colour fixation.

Dye Concentration (% owf)	Colour Fixation Agent to Cotton Weight Ratio (g/g)
0.1	0.06
0.5	0.06
1.5	0.08
2.5	0.09
3.5	0.09

**Table 4:** Concentration of dye (% owf) for preparing colour mixtures.

Dyeing Methods	Sample	Red	Yellow	Blue
Conventional water dyeing	Sample 1 (Standard 0.3%)	0.1	0.1	0.1
	Sample 2 (Standard 1.5%)	0.5	0.5	0.5
	Sample 3 (Standard 3.0%)	1	1	1
Reverse micellar dyeing	Sample 4 (Standard 0.3%)	0.1	0.1	0.1
	Sample 5 (Standard 1.5%)	0.5	0.5	0.5
	Sample 6 (Standard 3.0%)	1	1	1

**Table 5:** Colour matching recipes.

Formulae	Colour	Samples		
		Sample 1 (0.3%)	Sample 2 (1.5%)	Sample 3 (3%)
Standard sample (obtained from simulated dyeing)	Yellow	0.10	0.50	1.00
	Blue	0.10	0.50	1.00
	Red	0.10	0.50	1.00
Conventional Water-based Dyeing	Yellow	0.10 (0.00)	0.50 (0.00)	0.88 (−0.12)
	Blue	0.09 (−0.01)	0.44 (−0.06)	0.76 (−0.24)
	Red	0.08 (−0.02)	0.44 (−0.06)	0.83 (−0.17)
Octane-assisted Reverse Micellar Dyeing	Yellow	0.11 (+0.01)	0.56 (+0.06)	1.06 (+ 0.06)
	Blue	0.11 (+0.01)	0.53 (+0.03)	0.91 (−0.09)
	Red	0.09 (−0.01)	0.54 (+0.04)	1.10 (+0.10)

Positive number (higher than known concentration used in Section 2.6); Negative number (lower than known concentration used in Section 2.6).

**Table 6:** Δ*E* results. Note: Δ*E* denotes colour difference; *L* is lightness; *a* refers to the colour component of red and green; and *b* refers to colour component of yellow and blue.

Concentration	*L*_water_	*a*_water_	*b*_water_	*L*_octane_	*a*_octane_	*b*_octane_	Δ*E*
0.3%	68.17	6.42	4.02	67.72	6.42	4.50	0.66
1.5%	45.27	8.82	4.49	45.39	8.76	4.12	0.39
3.0%	36.57	9.66	3.89	36.88	9.45	3.59	0.48
